# Medical Education in Alcohol and Other Drugs: Curriculum Developement for Primary Care

**Published:** 1994

**Authors:** Catherine E. Dubé, David C. Lewis

**Affiliations:** Catherine E. Dubé, Ed.D., is a clinical assistant professor of community health and medical education and the curriculum development coordinator at the Center for Alcohol and Addiction Studies, Brown University, Providence, Rhode Island. David C. Lewis, M.D., is a professor of medicine and community health, Donald G. Millar Professor of Alcohol and Addiction Studies, and director of the Center for Alcohol and Addiction Studies, Brown University, Providence, Rhode Island

## Abstract

Over the past twp decades, physician education about alcohol and other drug abuse has progressed from teaching guides to full-blown curricula that focus on teaching medical students skills that are critical to the successful diagnosis and treatment of alcohol abuse. Despite these advances in teaching materials and techniques, integration of substance abuse curricula into primary care medical education has been slow at best.

Significant progress has been made during the past two decades in the development of curriculum materials for medical education in the field of alcohol and other drugs. The development of these materials has followed various trends in medical education, from slide-lecture-style didactic teaching^1^ to competency-based instruction and learner-centered and problem-based learning.

Over time, curricula in the alcohol and other drug abuse field have reflected a change in medical education, away from the biomedical model and toward the biopsychosocial model of care. This has been prompted by a host of factors, including the emergence of a primary care specialty, a movement toward the family practice model, a greater emphasis on holistic medicine, and a better understanding of the mind-body connection.

This article reviews the evolution of alcohol and other drug abuse medical education during the last two decades and presents current and future trends in learning.

## Curriculum Development During the Last Two Decades

Published curricula in the substance abuse field date back to the career teacher project, which began in 1971 and continued through 1983. As an outgrowth of this program, Hostetler (1982) published a methodology guide for teaching about alcohol and other drug abuse (table 1). This guide identified and described many of the newer methodologies for medical education, including interactive discussion, role play, and patient management problems. It also provided curriculum objectives for teaching.

### Developing a Structured Format

In 1984, the Commonwealth Harvard Alcohol Research and Teaching (CHART) program (Barnes et al. 1984) published an important curriculum that included detailed teaching outlines and an annotated bibliography. The teaching material was content oriented, used a seminar format, and provided some basic guidance on teaching clinical techniques and approaches. This new orientation toward clinical techniques and skills was significant because most teaching at that time focused only on content.

### Expanding the Focus

In 1987, the Society of Teachers of Family Medicine (STFM) published *The Family Medicine Curriculum Guide to Substance Abuse* (Liepman et al. 1987), which provided extensive reference and background information in a variety of areas, including pharmacology, pathophysiology, detoxification, prevention, and substance abuse in the family.

Learning objectives were listed for each of 10 teaching sessions. Teaching “hints” also were provided, including references for support material and ideas about laboratory experiments, films, and case presentations.

### Biomedical-Based Models

These models focus on the physical and medical characteristics of a disorder.

In 1981 and 1982, Project CORK (named for the developers), out of Dartmouth College, developed seven instructional units in a series called “The Biomedical Aspects of Alcohol Use.” The units contained more than 40 slides each, with accompanying text, and addressed topics such as pharmacology, alcohol and the liver, and alcohol and pregnancy. Three more units were published in 1989, featuring medical complications, abuse and dependence, and Native American use. A new unit, only on cocaine, is scheduled for release this year (VanWart personal communication, March 1994).

### Biopsychosocial-Based Models

In contrast to biomedical models of disease, biopsychosocial models take into consideration the psychological aspects of a disorder as well as social settings, such as the patient’s family, living arrangements, and resources.

#### Learner-Centered Models

In the early 1980’s, the Society of General Internal Medicine’s task force on medical interviewing (now the American Academy on Physician and Patient) began offering an innovative faculty development course that used a learner-centered model. This model included role play, small group process, personal awareness, and application/feedback techniques. The work of the task force reflected a movement in medicine away from the biomedical model of illness and medical care toward a more integrated biopsychosocial model, which was particularly applicable to teaching in the alcohol and other drug abuse arena. The course also advanced a skill-based approach to teaching and learning medical interviewing based on the work of Lipkin and colleagues (1985).

#### Competency-Based Models

Originally described by Davies (1973) and later by Mager (1988), competency-based instruction can be defined as teaching and learning designed specifically to produce skills and behaviors critical to the competent practice of medicine. Only key content related to competencies is included and is integrated with the attitudes and skills that build competency, rather than taught in isolation.

The Association for Medical Education and Research in Substance Abuse held a consensus conference in November 1985, known as the Annenberg Consensus Conference, to identify minimal competencies for physicians in the area of alcohol and other drug abuse. Competencies were identified based on clinical skills and behaviors that represented minimal qualifications for practicing physicians. Results of this seminal work formed the foundation for curriculum development for years to follow (Lewis and Niven 1985; Lewis 1990).

### Putting Programs Into Practice

In 1986, the National Institute on Alcohol Abuse and Alcoholism (NIAAA) and the National Institute on Drug Abuse (NIDA) jointly sponsored the Curriculum Models Program, which funded nine curriculum development projects in seven medical schools and one STFM project.

The objectives of this initiative were to move from the conceptual to the practical by (1) providing methods of integrating alcohol and other drug abuse education into primary care education, (2) employing new approaches to faculty development for alcohol and other drug abuse teaching, and (3) developing and pilot testing new curriculum materials.

#### Specialty-Specific Curricula

Some curricula were developed in terms of a specialty, such as a pediatric and an internal medicine curriculum project, both at the Johns Hopkins University, and a family medicine curriculum project at STFM.

#### Integrated Curricula

Other projects, such as those at the University of Virginia, Vanderbilt University, the Medical College of Virginia, and Brown University, employed an integrated approach. These programs included materials appropriate for all primary care specialties. Five of the nine program curricula were published. Two model curricula developed through this program are Project Alcohol and Drug Education for Physician Training (ADEPT) and Project Substance Abuse Education for Family Physicians (SAEFP). These curricula are described briefly below.

### A Closer Look at ADEPT and SAEFP

One of the integrated curriculum models, Brown University’s Project ADEPT, was the first model to address skills training in screening, assessment, and referral of alcohol and other drug problems appropriate to all primary care specialties. Teaching tools included videos, outlines, visuals for overheads or slides, handouts, case studies, and clinical application exercises.

A total of five volumes of Project ADEPT curriculum materials were published from 1989 to 1994 (Dubé et al. 1989*a*,*b*, 1990; Dubé and Lewis 1994*a*,*b*). Each volume addresses priorities in a different area: volume I covers basic clinical skills from diagnosis to treatment; volume II addresses special topics, including complications and comorbidities, the obstetrics/gynecology patient, nicotine dependence, and adolescents; volume III addresses the special considerations related to HIV and alcohol and other drug abuse behaviors; volume IV focuses on children of alcoholics and the elderly; and volume V offers a teaching program aimed at enhancing cultural competence and cross-cultural care.

The STFM’s Project SAEFP curriculum package used a faculty development model in its dissemination process (Project SAEFP executive committee and working group 1990). It was through this process that materials initially were disseminated to faculty around the country.

SAEFP conducted 10 5-day faculty development workshops at various locations around the United States during a 4-month period in 1990. These workshops were designed to reach physician faculty in every federally funded family medicine residency program in the country, enhance their clinical and teaching skills, and offer them a set of teaching tools that would help them provide structured didactic teaching sessions to their residents.

Teaching tools for SAEFP curriculum included goals, objectives, teaching strategies, lecture notes, summary tables, detailed background information, and visuals for slides or overheads.

### Curriculum Evaluation

The vast majority of curricula have been evaluated through face validity (i.e., expert and peer review) and pilot-testing techniques. Research on the effectiveness of specific curriculum packages is rare, and changes in teaching techniques often are based on faculty preference. Additionally, few dissemination studies have been conducted to determine the effects any one curriculum package may have had on teaching in the field of alcohol and other drug abuse. However, evidence of successful dissemination and acceptability of curriculum materials can be gleaned from distribution statistics.

For example, more than 1,000 volumes of Project ADEPT curriculum materials have been disseminated nationally and internationally during the past 5 years, with volumes IV and V newly released in July 1994. Further evidence of the acceptability of these materials comes from more than 20 requests on file to reprint or reproduce various aspects of Project ADEPT in coursebooks; texts; manuals; curricula; and technical bulletins for doctors, nurses, physicians assistants, and other primary care providers. Reprinting or reproducing of materials for typical teaching purposes cannot be measured because such duplication is specifically encouraged in the instructor’s guide, and no formal permission is required. A study of the dissemination patterns of each of the major curriculum packages for alcohol and other drug abuse training indirectly would provide useful insights into the impact such programs have had on teaching in the field.

### Measuring Change

Despite significant contributions to curricula in the alcohol and other drug field during the past 10 years, measurable changes in medical education continue to be slow.

In 1991, the Physician Consortium on Substance Abuse Education published a policy report stating that “substance abuse education and training for all levels of medical education is markedly deficient” (Lewis and Faggett 1991, p. 1).

The report recommended that medical education programs focus on altering physicians’ actual practice behavior rather than teaching general knowledge about substance abuse. Specific recommendations stressed the importance of faculty development, community-based training, and multidisciplinary approaches.

In a national curriculum survey, Fleming and colleagues (1994) found that the mean number of teaching units reported in a sample of 126 U.S. medical schools doubled—increasing from 3.5 units in 1986 to 7.4 units in 1992. However, only eight schools reported required (as opposed to elective) course work in the alcohol and other drug abuse area.

## Current Trends

### Competency-Based Curriculum

During the past 8 years, medical curricula in many settings have been evolving toward a competency-based model. Because of vast amounts of medical information and limited teaching time, medical educators have been forced to be more efficient, more directed, and more focused. In addition, competency-based approaches allow for greater efficiencies in teaching.

The process of developing competency-based instruction begins with a job analysis and expert review of practice behaviors considered to be important to the accomplishment of desirable job tasks. Curriculum designers must prioritize and define these behaviors and detail the knowledge, skills, and attitudes needed to perform each behavior (for a detailed description of the design process, see the box, p. 151).

**Developing a Competency-Based Curriculum for Alcohol and Other Drug Abuse**

The first step is to identify and examine physician practice behaviors important to the care—prevention, diagnosis, assessment, treatment, and ongoing care—of patients with alcohol and other drug problems.

These behaviors might include such items as:

- Taking an alcohol and other drug use history
- Using diagnostic criteria to assess the severity of an alcohol or other drug use problem
- Developing treatment plans for patients with alcohol and other drug problems
- Referring a patient to treatment
- Following up with patients during and after treatment.

Expert practitioners and teachers can help develop an exhaustive list of practice behaviors and then prioritize the list. To help, they can use the following scale:

**Table:** 

Priority	Definition
1	Absolutely essential: would be malpractice to leave out.
2	Very important: should include if at all possible.
3	Can be left out if teaching time runs out.

Each of the top or second priority items on the list is further detailed so that an extensive list of component parts for each behavior is developed. Component parts always include knowledge, skills, and attitudes. For example, component parts of the item “taking an alcohol and other drug use history” mentioned above might include:

Knowledge

- Diagnostic criteria for abuse and dependence
- Terminology: drugs of abuse
- Natural history of alcohol and other drug abuse problems

Skills

- Asking about past use patterns
- Applying the diagnostic criteria
- Asking about previous attempts to quit
- Asking about previous treatment experiences
- Assessing the patient’s history

Attitudes

- Unconditional positive regard for the patient
- Caring, helpfulness, and empathy for the patient’s experience(s).

Developers can then examine the highest priority behaviors in detail. To examine fully these behaviors, they must define the role of the physician and answer the question, “What would a competent physician do to accomplish this task?” Answers to this question can be obtained from concerned and enlightened teachers in the field and by interviewing and observing an “expert” performing the skill (e.g., counseling adolescents about alcohol behaviors). The practice behavior can then be documented and broken down into its component parts so it can be taught and learned. Component parts might include such activities as:

- Bringing up the topic of drinking behaviors among peers and family
- Assessing the patient’s current drinking behaviors
- Determining context and setting of current drinking behaviors
- Developing a common understanding of the risks of drinking
- Identifying motivational factors for changing risky behaviors
- Linking benefits of changing drinking behaviors to motivational factors
- Negotiating change
- Following up.

Each of these components can then be further defined for review by experts, and additional knowledge and attitudes related to these more detailed behaviors can be identified to be sure that the task can be fully, adequately, and systematically taught.

Designing a competency-based curriculum is time consuming, but the result is a roadmap with well-identified priority areas, such as the important skills of detection and diagnosis of alcohol and other drug problems. A wide range of related areas, such as case discussion points and clinical application exercises, are included should there be additional teaching time. The MD 2000 curriculum project at Brown University and all Project ADEPT programs rely on a similar competency-based design. The strength of this approach is its systematic and analytical process, which yields practical and useful teaching materials. This is particularly important in the field of alcohol and other drugs because teachers rarely have sufficient time in the medical curriculum to teach the important clinical skills and behaviors in this field.

The one drawback is the time and resource drain that occurs during the front-end analysis (i.e., audience analysis, job analysis, and behavioral outcome analysis) and development phases. Furthermore, the focus on clinical practice skills and behaviors calls for teaching methods that depart from traditional didactic formats and often requires additional faculty training and development.

### Problem-Based Learning

Many medical schools^2^ have been moving toward a problem-based curriculum that emphasizes case studies and research over content. This move has been prompted by a desire to be more learner centered and to impart lifelong learning skills.

Compared with traditional teaching approaches, problem-based teaching requires significantly different instructional aids and tools. Instead of a syllabus, students in a problem-based environment require case materials. Teachers need background information and resources that allow them to provide guidance to students in their quest for information. Classes, at the least, need group facilitation guidelines, clear objectives and goals for problem-based work, clear guidelines for evaluation, and an organized series of intriguing cases that also stimulate important teaching activities (including discussion, research, presentations, and further inquisition).

Problem-based learning offers students the opportunity not only to learn about a topic but also to research the topic and evaluate, integrate, and present their findings. Students exposed to problem-based learning exhibit significant enthusiasm for their work because they present their own findings and they teach and learn from each other. A wide variety of topics typically are presented after case research. These topics often extend beyond what a typical lecturer might include.

The drawback of problem-based learning is the time, faculty, and resource requirements. The student-faculty ratio for a traditional problem-based group may be as small as 6 to 1. Faculty also need training in this technique to make it work. Without adequate training, faculty may regress to didactic, teacher-directed, content-oriented models. Also, case discussion prior to student research takes as long as one lecture on a given topic. Furthermore, debriefing sessions, in which all students have an opportunity for presentation and discussion of findings, can easily take the time of two didactic teaching sessions on the same topic.

The problem-based format is well suited to teaching about alcohol and other drug problems. Because these problems include biopsychosocial, spiritual, and political elements, any case will be rich with research possibilities. However, because this method requires a significant commitment of time by faculty members, it may be difficult to implement this ideal approach.

### Community-Based Teaching

Medical schools, government-funding agencies, and policy leaders in health care reform currently are promoting community-based medical education. This shift in focus from a university-based and hospital-based setting to a community-based one is a result of the need to emphasize primary care in medical education and the need to broaden medical student experience.

Community-based medical education provides training in a variety of settings with a variety of patients and can offer a training experience that is closer to practice in the “real world” than training provided only in an academic medical center. However, much like problem-based teaching, it is resource intensive.

Because training increasingly will occur in community settings not currently affiliated with an academic medical center, programs will have to train a new set of faculty and preceptors in clinical supervision and teaching techniques. Community-based faculty will need materials that help structure the field-training experience and provide students with appropriate learning activities and opportunities for research, reflection, and feedback. A curriculum package for community-based teaching would provide time-efficient tools and methods for supervision, precepting, and consultation with students as well as means for satisfying educational standards for the experience. Without such an approach to community-based teaching, the field will move back to the old apprenticeship model, which is an inefficient approach to physician training. Thus, tools for community-based learning will help standardize education, but they also will provide criteria for the quality of teaching and learning as well as provide focus and meaning to educational experiences.

Community-based medical education has great appeal to medical educators in the field of alcohol and other drug abuse. It provides a unique opportunity to recruit students into the treatment centers, to allow them the opportunity to learn about the treatment and recovery process, and to give them the chance to develop positive relationships with addicted and recovering patients in a medical environment. The field could benefit from a community placement curriculum package that provides a structure and framework for such an experience.

## Future Trends

Finding the appropriate methods and content necessary to train competent practicing physicians depends on the definition of their appropriate roles and responsibilities. The role of the physician is not stagnant but dynamic and affected by many factors, including influences on the field of medicine, market forces and consumer demand, changing national culture and climate, advances in science and practice, and changes in reimbursement systems and national policy. Currently, the most significant factor influencing the future of the physician’s role is impending health care reform.

## Health Care Reform

Health care reform will be a major impetus to encourage physicians to diagnose and treat alcohol and other drug abuse problems early, before costly accidents and acute and chronic medical illnesses supervene. It is now anticipated that some degree of alcohol and other drug abuse treatment will be included in the basic core benefit. Preventive clinical services also will receive more support as will increased training for primary care physicians—the so-called gatekeepers of the new system. Furthermore, managed care will force explicit diagnoses and patient matching to acceptable treatment modalities.

Spurred on by the health care reform debate, the American Medical Association (AMA), through the urging of their House of Delegates at their New Orleans meeting in December 1993, passed the following recommendations:

- That the AMA encourage all physicians, particularly those in primary care fields, to undertake education in treating alcohol and other drug abuse.
- That the AMA direct its representatives to appropriate residency review committees (RRC’s) to ask the committees on which they serve to consider requiring instruction in the recognizing and managing of alcohol and other drug abuse. Those RRC’s that already require such instruction should consider greater emphasis for this subject.
- That the AMA encourage treatment of alcohol and other drug abuse as a subject for continuing medical education.
- That the AMA affirm that many physicians in fields other than psychiatry have graduate education and experience appropriate for the treatment of alcohol and other drug abuse, utilization review, and other evaluation of such treatment, and should be entitled to compensation for performing those services.

### Incentives for Substance Abuse Training

Assuming that health care reform results in a capitated health care system (i.e., one that requires physicians to charge a standard fee up front for each patient as opposed to a fee-for-service system), it will be in the interest of both purchasers and providers of service to address alcohol and other drug problems routinely because untreated alcohol and other drug abuse is so disabling and costly. The development of value, quality, and cost consciousness would drive the system to provide more routine and effective substance abuse services. These incentives, however, would make the shortfall in substance abuse training of physicians even more apparent and the need for new training incentives and standards a priority.

The danger is that the system may change so rapidly under health care reform that physicians will not be ready to participate in that system.

### Faculty Training

Trained faculty to teach the skills related to alcohol and other drug use problems in medical schools and other health care professions continue to be needed. With the emergence of new nontraditional contexts and formats for teaching in both academic and community-based settings, greater emphasis should be put on faculty development.

The faculty development program grants, initially administered by NIAAA and NIDA and now administered by the Center for Substance Abuse Prevention, in part address a lack of faculty with basic skills. The grants aim to develop a cadre of skilled faculty with professional identification in the field of alcohol and other drug abuse in each participating institution. However, with this program winding down, the number of core faculty capable of teaching basic skills at many medical schools remains low.

## Conclusion

Our most valued resources in academic medicine are hard-working, dedicated faculty, including researchers, clinicians, and educators. Providing these teachers with the means, methods, and tools necessary for excellence is where future curricular design and development and continued faculty development programs can help. Continued attention to curricular development and faculty development in alcohol and other drug abuse can help maintain the gains made in the field over the past two decades and help continue to promote essential alcohol and other drug abuse teaching in U.S. medical schools.

## Figures and Tables

**Table 1:** Summary of Alcohol and Other Drug Abuse Curricula for Physicians

Curriculum	Contents	Strengths	Weaknesses	Availability
Alcohol and Drug Abuse Teaching Methodology Guide for Medical Faculty (1982) Jeptha Hostetler, Ph.D.	Teaching in the Medical Setting Curriculum Objectives Teaching Methodologies	Provides background information and suggestions for teaching activities.	No specific teaching sessions described.	Not available.

Commonwealth Harvard Alcohol Research and Teaching Program (CHART) (1984) Robin Barnes, M.D., et al.	Epidemiology Detection of Alcoholism Presenting the Diagnosis Treatment and Management Planning for Disposition Alcoholics Anonymous (AA) Development of Curriculum	Provides detailed teaching outlines and an annotated bibliography.	No supporting materials provided.	Not available.

Family Medicine Curriculum Guide to Substance Abuse (1987) Michael Liepman, M.D., et al.	Pharmacology of Commonly Abused Drugs Pathophysiology of Ethanol Abuse Finding Substance Abusers Motivating Change Detoxification Chemical Dependency in the Family Family Physician at Risk Strategies for Curricular Change	Includes reference and background information, learning objectives, and teaching hints.	No detailed teaching outlines, instructions, or tools are provided. Difficult to read.	For ordering information call: (816) 333–9700 ext. 4510 The Society of Teachers of Family Medicine 8880 Ward Pkwy. P.O. Box 8729 Kansas City, MO 64114 *NOTE: This publication is on clearance and will soon be out of print.*

The Biomedical Aspects of Alcohol Use (1982, 1989, 1994) Jean Kinney, M.S.W., et al.	Biochemistry, Pharmacology and Toxicology The Liver Hematologic Complications The Alimentary Tract and Pancreas Pregnancy and the Fetal Alcohol Syndrome Endocrine and Metabolic Effects Neurological Complications Medical Complications Alcohol Use, Abuse and Dependence Native American Alcohol and Substance Use Cocaine	Provides more than 500 teaching slides with commentary and supporting booklets.	Costs approximately $1,000. Limited to slide-lecture format.	For ordering information call: (800) 432–8433 Project Cork Institute Dartmouth Medical School Suite 2F 14 South Main St. Hanover, NH 03755–2015

Project ADEPT				
Volumes I and II Core Modules and Special Topics (1989) Catherine Dubé, Ed.D., et al.	Introduction and Overview Prevention Assessment and Diagnosis Presenting the Diagnosis Treatment Adolescent Substance Use OB/GYN Patients Health Professional Impairment Nicotine Dependence Complications and Comorbidities	Provides outline and supporting materials for each teaching session. Is competency based. Focuses on core clinical skills.	Requires training in the field of alcohol and other drug abuse and in small group teaching techniques.	For ordering information call: (401) 863–7791 Brown University Center for Alcohol and Addiction Studies Box G–BH Brown University Providence, RI 02912
Volume III AIDS and Substance Abuse (1991) Catherine Dubé, Ed.D., et al.	Overview Patient Counseling Screening Counseling About HIV Testing Followup Counseling Counseling About Risk Behaviors Video Teaching Guide	Provides small, manageable teaching units. Is competency based. Focuses on clinical skills.	Requires specific training for faculty. Assumes previous knowledge about HIV.	See above.
Volume IV Special Populations (1994) Catherine Dubé, Ed.D., et al.	Identification and Referral of Children of Alcoholics Alcohol and Other Drug Problems in the Elderly	Provides a complete teaching unit with video from the Children of Alcoholics Foundation and four units of instruction on the elderly.	Requires specific training for faculty.	See above.
Volume V Race, Culture and Ethnicity: Addressing Alcohol and Other Drug Problems (1994) Catherine Dubé, Ed.D., et al.	Introduction and Overview Cultural Self-Awareness Exercise Cross-Cultural View of Health Care and Alcohol and Drug Use Cross-Cultural Stimulation Exercise Risk Factors, Stress and Coping Screening and Intervention Skills Practice and Application	Provides supporting materials including video and self-awareness and experiential teaching exercises. Avoids cultural stereotyping.	Depends strongly on quality of instructors. Simulation exercise requires significant preparation. Little culture-specific information.	See above.

Project SAEFP Substance Abuse Education for Family Physicians (1990) Michael Fleming, M.D., M.P.H., et al.	Whose Problem Is It? Attitudes and Values Magnitude of the Problem Interviewing and Identification Treatment Detoxification Initial Management 12-Step Programs Drug Abuse Physician Impairment Family Issues Educational Strategies	Provides lecture notes, background information, and teaching slides. Offers multiple teaching strategies.	Heavy emphasis on lecture techniques in some units. Requires trained faculty.	For ordering information call: (816) 333–9700 ext. 4510 The Society of Teachers of Family Medicine 8880 Ward Pkwy. P.O. Box 8729 Kansas City, MO 64114

## Footnotes

1 For a definition of this term and other terms used in this article, see the glossary on p. 152.

2 These schools include Harvard Medical School, the University of Newcastle in Australia, McMaster University in Canada, Ben Gurion University in Israel, and the University of Limburg in the Netherlands.

## GLOSSARY

Application/feedback techniques: Teaching techniques that allow students to try new skills quickly. Often a trained instructor is available to provide timely and accurate feedback on the effectiveness of skills students employ.
Biomedical model: An orientation to patient care that stresses the disease process on the cellular and organ levels. Compare with *biopsychosocial model*.
Biopsychosocial model: An orientation to patient care that takes into consideration not only disease but also the patient’s social setting (e.g., family, support systems, living situation, and resources) and the psychological aspects of illness. This orientation is particularly applicable to diseases such as addiction, which have significant social and psychological components. Compare with *biomedical model*.
Competency-based instruction: Instruction designed to reflect real and important clinical practice behaviors that are employed routinely by a competent practitioner.
Community-based teaching: Teaching offered in the context of community health care settings such as community health centers, physicians’ offices, senior centers, or urban or rural health clinics.
Content-oriented teaching: Teaching that focuses on knowledge and facts. Using a *didactic* format, this traditional method of teaching is not effective in teaching skills, attitudes, or complex behaviors.
Curriculum: In this article, a curriculum is defined as an organized set of teaching or learning activities structured to address a given topic fully. All the curricula referred to have been documented and published in a curriculum package that includes related teaching instructions and tools.
Curriculum tools: Helpful tools for the instructor that are included in a curriculum package. These tools might include checklists for teaching preparation, handouts for students, videos for discussion, slides or overhead transparencies to use in teaching, exercises and cases for session activities, and instructors’ outlines.
Didactic teaching: Instructor-led teaching that often uses a slide-lecture format and usually is limited to imparting facts and information rather than skills or behaviors.
Learner-centered teaching: A teaching technique that allows students to participate actively in setting educational goals and selecting learning strategies. This method is in contrast to teacher-centered techniques, where goals and strategies are dictated by the instructor.
Personal awareness: A group process in which interpersonal aspects of patient care, feelings regarding job and profession, and personal struggles in these areas are discussed. Personal awareness groups often employ group therapy techniques.
Problem-based format: A teaching method that uses cases to trigger self-study or group-study experiences for students. Presented with a case, students are led by the instructor through a process whereby the problem case is defined, unanswered questions are identified, and areas for investigation are selected. Students investigate and report back to the group on areas of investigation.
Role play: An instructional exercise often used in skills teaching. Participants in the teaching session take on the role of doctor or patient so that patient interactions can be practiced. Effective role play techniques include the instructor’s facilitation of the interaction (i.e., using stop-start techniques; swapping roles between doctor and patient; introducing new doctors, family members, or other characters; and adding changes and challenges to the case). The instructor also provides effective, timely, behaviorally based, and accurate feedback on performance and facilitates both the performer’s self-evaluation of skills and the peer evaluation of the interaction.
Skills-based teaching: A teaching technique that stresses the acquisition of skills rather than memorization of facts. Techniques include teaching exercises appropriate for the learning and practice of skills. For example, for patient interaction skills, appropriate exercises include *role play*, simulated patient interviews, and actual patient interviews.
Small group process: An interactive method of teaching that employs discussion among 5 to 12 students to examine problems, develop strategies, practice solutions, enhance insights, and address attitude changes. Effective small group process often will include a video trigger, case, or problem for discussion and will employ *learner-centered* or *problem-based* techniques.
